# Catalytic Oxidation of CO and Soot over Ce-Zr-Pr Mixed Oxides Synthesized in a Multi-Inlet Vortex Reactor: Effect of Structural Defects on the Catalytic Activity

**DOI:** 10.1186/s11671-016-1713-1

**Published:** 2016-11-10

**Authors:** Samir Bensaid, Marco Piumetti, Chiara Novara, Fabrizio Giorgis, Angelica Chiodoni, Nunzio Russo, Debora Fino

**Affiliations:** 1 Department of Applied Science and Technology, Politecnico di Torino, Corso Duca degli Abruzzi 24, 10129 Torino, Italy; 2 Center for Sustainable Futures - CSF@POLITO, Istituto Italiano di Tecnologia, Corso Trento 21, Torino, 10129 Italy

**Keywords:** Ceria, Zirconia, Praseodymia, Dopants, Structural defects, CO oxidation, Soot oxidation

## Abstract

In the present work, ceria, ceria-zirconia (Ce = 80 at.%, Zr = 20 at.%), ceria praseodymia (Ce = 80 at.%, Pr = 20 at.%) and ceria-zirconia-praseodymia catalysts (Ce = 80 at.%, Zr = 10 at.% and Pr = 10 at.%) have been prepared by the multi-inlet vortex reactor (MIVR). For each set of samples, two inlet flow rates have been used during the synthesis (namely, 2 ml min^−1^_,_ and 20 ml min^−1^) in order to obtain different particle sizes. Catalytic activity of the prepared materials has been investigated for CO and soot oxidation reactions. As a result, when the catalysts exhibit similar crystallite sizes (in the 7.7–8.8 nm range), it is possible to observe a direct correlation between the O_v_/F_2g_ vibrational band intensity ratios and the catalytic performance for the CO oxidation. This means that structural (superficial) defects play a key role for this process. The incorporation of Zr and Pr species into the ceria lattice increases the population of structural defects, as measured by Raman spectroscopy, according to the order: CeO_2_ < Ce_80_Zr_20_ < Ce_80_Zr_10_Pr_10_ < Ce_80_Pr_20_. On the other hand, the presence of zirconium and praseodymium into the ceria lattice does not have a direct beneficial effect on the soot oxidation activity for these catalysts, in contrast with nanostructured ones (e.g., Ce–Zr–O nanopolyhedra, Ce–Pr–O nanocubes) described elsewhere (Andana et al. Appl. Catal. B 197: 125–137, 2016; Piumetti et al., Appl Catal B 180: 271-282, 2016).

## Background

During the last few decades, ceria-based catalysts and related materials have extensively been investigated, thanks to their interesting redox properties that allow rapid oxygen intake-uptake [1–3]. Many studies have proven the beneficial role of ceria in catalyzing soot and CO oxidations [4–10]. In soot combustion, whose non-catalytic oxidation occurs at temperatures above 550 °C [11], a good contact between the catalyst and soot particles is necessary to considerably reduce the oxidation temperatures; this is normally attained by tuning ceria morphology at the nanoscale size [7, 8, 12–14]. Moreover, the ability of catalysts to activate oxygen species (O*) is strictly necessary for this oxidation reaction. On the other hand, in CO oxidation, the oxygen storage capacity (OSC) plays a key role since this reaction catalyzed by ceria takes place through the Mars-van Krevelen (MvK)-type mechanism [3–5, 15]. According to this mechanism, indeed, oxygen vacancies (O_v_) are created during the CO oxidation at catalyst surface, followed by oxygen refilling from the bulk phase. The incorporation of metal dopants perturbs the material structure, creating defects that may take form as vacancies or interstitial defects. The latter are usually the prerequisite of enhanced the oxidation activity and thermal stability [3, 16–18]. Among the various elements used as dopants for ceria catalysts, zirconium and praseodymium have received a major interest over the last few years [6, 12, 16, 19]. Zr or Pr species embedded into the ceria lattice promote the formation of crystallographic defects [6, 12]. As a whole, structural defects in metal oxide catalysts are highly desirable since they can enhance the number of oxygen vacancies (O_v_) and hence the electronic properties are favored, namely the mobility of electrons and negatively charged ions in the solid framework [16]. Moreover, our recent study with Ce–Zr mixed oxides has shown that the use of non-reducible, isovalent Zr^4+^ improves both the oxidation activity and thermal stability of the ceria catalysts [6]. Conversely, the aliovalent praseodymium has been identified as a “redox cycle promoter” [12]; the presence of this metal, indeed, would favor the creation of Ce^3+^ species in the ceria [12].

The present study focuses on the synthesis of ceria-based catalysts doped with zirconium and praseodymium in a micro-fluidic device, designed and operated to control the size of the nanoparticles. The selected synthesis technique [20] is based on the preparation of an aqueous solution of metal precursors together with a concentrated sodium hydroxide solution (pH 13), which are fed to a mixing chamber, where they react leading to the precipitation of the nanoparticles. This synthesis technique is characterized by simple operations, it involves low-cost reagents and, instead of the conventional solution combustion synthesis technique [19] (which is carried out at 650 °C), is an ambient temperature precipitation. Moreover, this synthesis route is extremely useful in the perspective of continuous ceria-based particle production, with the specific purpose of controlling the particle diameter and obtaining reproducible results in terms of particle size distribution.

Micro-mixers with characteristic sizes from hundreds of micrometers to few millimeters are very appealing given the turbulent flow originated by flow instabilities, and the consequent turbulent mixing, that occur under particular operating conditions [21]. Relevant cases of such devices are the T-Mixer [20, 22], the Multi-Inlet Vortex Mixer (MIVM) [23, 24] and the confined impinging jets reactor (CIJR) [25]. These categories of mixers differ by the size of the mixing chamber, the number of inlets, the position of the inlets (axially aligned or unaligned) and the size of the outlet channels with respect to the inlet ones. In our research activity, a Vortex-shaped micro-mixer, called multi-inlet vortex reactor (MIVR), was employed to synthesize ceria-based catalytic nanoparticles with the possibility to tune the particle sizes according to the fluid-dynamics inside the reactor. More specifically, the predictions of spatial distributions of reactant concentrations, obtained through the fluid-dynamic computation of the micro-mixer internal flow field, indicated the mixing effectiveness of the reactants at the operating conditions selected for the syntheses [26].

The sensitivity of the primary nanoparticles to the synthesis operating conditions was investigated, as well as the effect that such particle distribution exerts on the catalytic activity towards CO and soot oxidation. Indeed, the complex interplay between the catalyst composition (ceria with Zr and Pr doping), primary particle distribution (with its effect on the specific surface area) and overall aggregate morphology (affecting the soot-catalyst contact) and the catalytic reaction (CO and soot oxidation) was tackled in the attempt to discriminate the dominant factors that concur to the overall catalytic activity.

## Methods

### Preparation of Samples

**Fig. 1:**
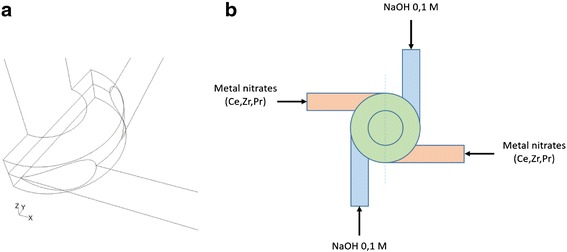
Schematization of the MIVR (**a** half-reactor view and **b** top view)

The customized geometry of the investigated MIVR designed for ceria-based catalysts production (Fig. 1a) is characterized by four tangential circular inlets, each one with an internal diameter of 1 mm, and a reaction chamber of 4 mm in diameter and 1 mm in height. The product is discharged in a 2 mm diameter channel, perpendicular to radial inlets. The four inlets were connected to four 20 ml syringes, and driven by a KD Scientific KDS220 infusion syringe pump. The syringes were filled in pairs with the two reactant solutions: a solution of metal precursors (Ce(NO_3_)_3_ · 6H_2_O, ZrO(NO_3_)_2_ · xH_2_O and Pr(NO_3_)_3_ · 6H_2_O all from Sigma-Aldrich) with a total metal concentration equal to 0.1 M, and solution of sodium hydroxide in water also 0.1 M (leading to a pH equal to 13). The solutions were fed to the four channels (Fig. 1b) alternating the metal precursors solution and the NaOH one, to maximize the mixing efficiency.

Pure ceria (denoted as Ce100), ceria-zirconia (denoted as Ce80Zr20; Zr atomic percentage = 20 %), ceria-praseodymia (denoted as Ce80Pr20; Pr atomic percentage = 20 %) and ceria-zirconia-praseodymia (denoted as Ce80Zr10Pr10; Pr, Zr atomic percentage = 10 %) were synthesized. When Ce100 was produced, the solution containing the metal precursor was 0.1 M in Ce(NO_3_)_3_ · 6H_2_O; on the other hand, when other metals were present, the concentration of 0.1 M was referred to all metals, being in molar ratios of the precursors the same as denoted in the final sample Ce_x_Zr_y_Pr_z_.

The two solutions were fed with two inlet flow rates: 2 and 20 ml min^−1^ in each of the four inlet channels (*D* = 1 mm). This leads to a Reynolds number in the chamber Re_c_ of 832 and 8320, respectively (*D*_*c*_ = 4 mm in this case). These two inlet flow conditions correspond, according to the computation of the MIVR fluid dynamics [26], to a laminar flow inside the reactor for 2 ml min^−1^ whereas a turbulent one for 20 ml min^−1^, with unsteady fluctuations of the flow. This flow regime difference has an impact on the primary particle distribution of the precipitated nanoparticles, whose mean diameter was estimated a posteriori through field emission scanning electron microscopy.

The outlet solution, which contains the nanoparticles, was collected in a beaker and maintained in agitation until the infusion was complete and the suspension was ready to be centrifuged (4000 rpm for 1 h). Afterwards, the resulting deposit was separated from the solution, washed with distilled water, and re-centrifuged. Finally, the solid was placed in a quartz filter (0.45 μm), under which a vacuum pump eliminated the residual aqueous solution permeating the powder. The collected solid was then calcined in an oven at 650 °C for 2 h, in air.

### Characterization of Samples

Powder X-ray diffractograms (XRD) of the samples were recorded using a X’Pert Philips PW3040 diffractometer with Cu Kα radiation, 2*θ* range of 20°–70° (angle step size at 0.02°) and a time per step of 0.2 s. International Centre of Diffraction Data (ICDD) was used for peak identification. The Scherrer’s equation (namely, *D* = 0.9*λ*/*b*cos*θ*) was used to estimate the crystallite size of the samples, where *λ* is the wavelength of the Cu K*α* radiation, *b* is full width at half maximum in radians, 0.9 is the shape factor for spheres, and *θ* is the diffraction peak angle. Specifically, the powder XRD analysis was carried out with an internal standard (highly crystallized CeO_2_ obtained at 900 °C for 24 h) and each measurement was done by considering three main peaks. Then, the mean and deviation standard values of crystallite size have been calculated.

N_2_ physisorption at −196 °C was performed to study the textural properties of the catalysts, such as the specific surface area and total pore volume (Micrometrics ASAP 2020 instrument). Removal of water and contaminants from the samples was done prior to analyses by heating the samples at 200 °C for 2 h. BET method was applied to calculate the specific surface area.

Field emission scanning electron microscopy (FESEM) has been performed with a ZEISS Auriga crossbeam, equipped with an OXFORD X-act Energy dispersive X-ray detector. Size distribution of the primary particles, i.e., the ones originated by single nuclei which then grow by solute diffusion from the solution to the particle surface, was derived for all samples from several representative FESEM images. In particular, FESEM imagery was used to measure the primary particle average diameter; around a hundred measurements were performed for each catalyst sample, thus reaching a representative evaluation of the primary particle overall distribution. Conversely, aggregate sizes were not measured (i.e., through light scattering techniques) since their size and structure cannot be controlled due to the aggregation phenomena and particle sedimentation occurring in the beaker that collects the suspension at the reactor outlet.

Transmission electron microscopy (TEM) has been performed with a FEI Tecnai F20ST, operating at 200 KV both in TEM and HRTEM modes, equipped with an EDAX Energy dispersive X-ray detector.

Raman measurements were performed using a Renishaw InVia Reflex micro-Raman spectrometer with laser excitations at 457.9 and 785 nm in backscattering light collection. The O_v_/F_2g_ value, representative of the oxygen vacancies density, was calculated as the ratio between the integrated areas of the O_v_ and F_2g_ Raman peaks obtained by averaging the spectra recorded on five different points of the sample. The regions for the integration were selected depending on the Raman shift of the peaks for each catalyst composition and excitation wavelength. The calculation for Ce_80_Zr_10_Pr_10_ at 785 nm is reported as an example:

$$ \frac{O_v}{F_{2\mathit{\mathsf{g}}}}=\frac{area\;{O}_v\left(505-660\;c{m}^{-1}\right)}{area\;{F}_{2\mathit{\mathsf{g}}}\left(320-505\;c{m}^{-1}\right)}=\frac{8.79}{39.35}=0.22 $$

### Catalytic Tests

CO oxidation reaction was carried out in a fixed-bed quartz reactor (U-type tube, 4 mm inner diameter) heated by a furnace. In a typical test, 0.1 g of powdered sample was inserted in the reactor. The temperature of reactor bed was detected by a K-type thermocouple, placed as close as possible to the bed. The test started by continuously flowing 50 ml min^−1^ gas containing 1000 ppm of CO and 50 %-vol air in N_2_ into the reactor. Meanwhile, the furnace heated up at a rate of 5 °C min^−1^ until complete CO conversion was reached. Non-dispersive infrared analyzers were used to record CO_x_ concentrations at the reactor outlet.

Soot oxidation tests were conducted in the same fixed-bed Quartz reactor. In a typical run, a reactor bed contained 5 mg of soot (Printex-U), 45 mg of powdered sample and 150 mg of silica. The bed was prepared by ball-milling the solid mixture at 250 rpm for 10 min to eventually attain “tight contact”. The test started by flowing 100 mL min^−1^ gas comprising of 50 %-vol of air and 50 %-vol of N_2_ to the reactor. The reaction temperature increased from 100 to 700 °C with 5 °C min^−1^ heating rate. The gaseous mixtures were analyzed via CO/CO_2_ NDIR analyzers (ABB). Temperatures corresponding to 10, 50, and 90 % conversion of either CO or soot (denoted as T10, T50, and T90 %, respectively) were taken as indices of the catalytic activity.

## Results and Discussion

### Textural and Structural Properties of the Catalysts

**Fig. 2:**
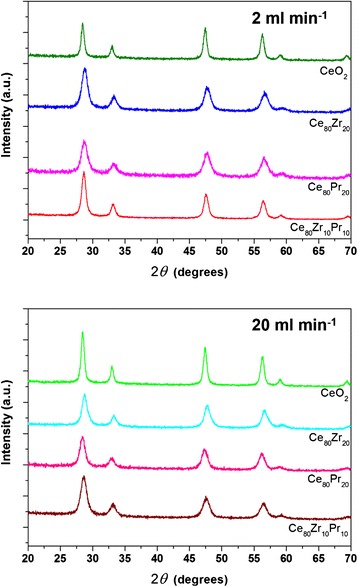
XRD patterns of the samples prepared at 2 ml min^−1^ (*top*) and 20 ml min^−1^ (*bottom*)

Figure 2 shows the XRD diffractograms of the prepared catalysts. The latter exhibit similar XRD patterns referring to cubic fluorite structure, marked by the presence of (111), (200), (220), (311), (222), and (400) planes [26]. No additional peaks due to the presence of either ZrO_2_ or PrO_2_ phases can be detected, thus confirming a good incorporation of the dopants into the CeO_2_ lattice for both binary and ternary oxides. Estimation through the Scherrer’s equation, using the (111) peak as reference, shows that catalysts prepared with inlet flow rates of 2 ml min^−1^ have bigger crystal sizes than their counterpart obtained at 20 ml min^−1^ (Table 1). A similar trend appears for the particle sizes estimated through the FESEM analysis. More precisely, the primary particles in the synthesis condition at 20 ml min^−1^ have an average diameter very close to the one of the crystallite. Moreover, the size distribution of the primary particles is very narrow; therefore, this occurrence applies to the majority of the primary particles. On the other hand, the primary particles at 2 ml min^−1^ differ from the crystallites in term of sizes, especially for the doped samples. It is possible that, in the latter case, the higher residence time in the reactor entail the coalescence of few crystallites to form a primary particle.

**Table 1:** Specific surface areas, crystallite sizes, and primary particle sizes of the prepared catalysts

Sample	MIVR single channel inlet flow rate = 2 ml min^−1^			MIVR single channel inlet flow rate = 20 ml min^−1^		
	SSA^a^ (m^2^g^−1^)	D^b^ (nm)	d_P_^c^ (nm)	SSA^a^ (m^2^g^−1^)	D^b^ (nm)	d_P_^c^ (nm)
CeO_2_	36	16.3 ± 0.4	17.9	43	14.5 ± 0.5	9.8
C_80_Zr_20_	55	8.7 ± 0.8	17.1	63	9.2 ± 0.3	8.1
Ce_80_Pr_20_	49	9.5 ± 0.6	16.4	61	10.4 ± 0.8	7.3
Ce_80_Zr_10_Pr_10_	37	10.2 ± 0.7	17.2	77	7.6 ± 0.5	8.1

^a^Specific surface areas calculated by the BET method

^b^Crystallite sizes measured via Scherrer’s equation

^c^Primary particle sizes estimated by FESEM analysis

**Fig. 3:**
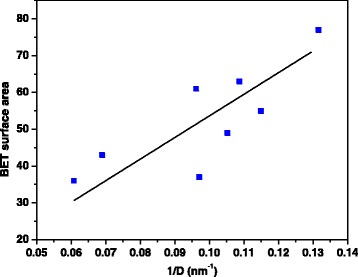
Correlation between the BET specific surface area and the inverse average crystallite size derived by the Scherrer formula

This finding is in agreement with the fact that lower residence times into the MVIR favor the formation of smaller particle sizes. The BET surface area, derived from N_2_ physisorption measurements at −196 °C are also reported in Table 1: the samples synthesized with inlet flow rates of 2 ml min^−1^ have lower surface areas than the counterpart obtained at higher flow rates. Moreover, the presence of the dopants into the ceria increases the specific surface area values for both synthesis conditions. The dependence of the specific surface area on inverse average crystal size is reported in Fig. 3. Since a quasi-linear correlation occurs, then the crystal shape of the catalysts can be considered approximately spherical (“unstructured” materials) [27].

**Fig. 4:**
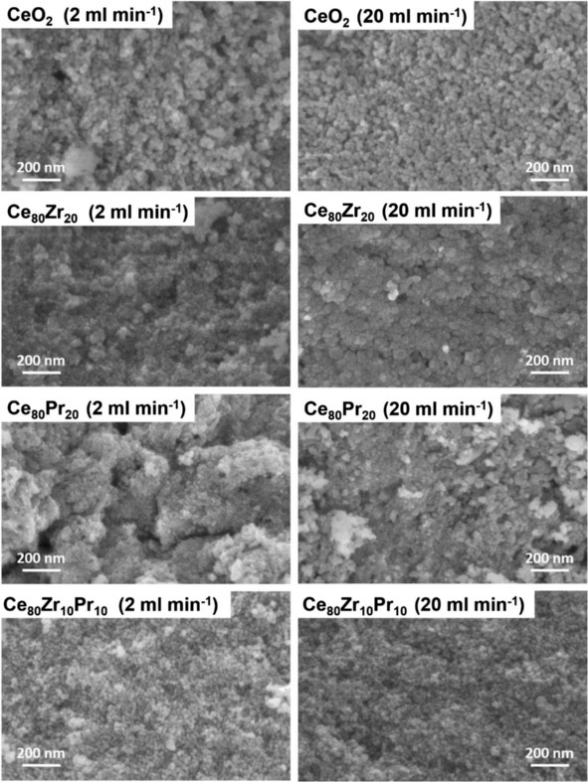
FESEM images of the prepared catalysts

**Fig. 5:**
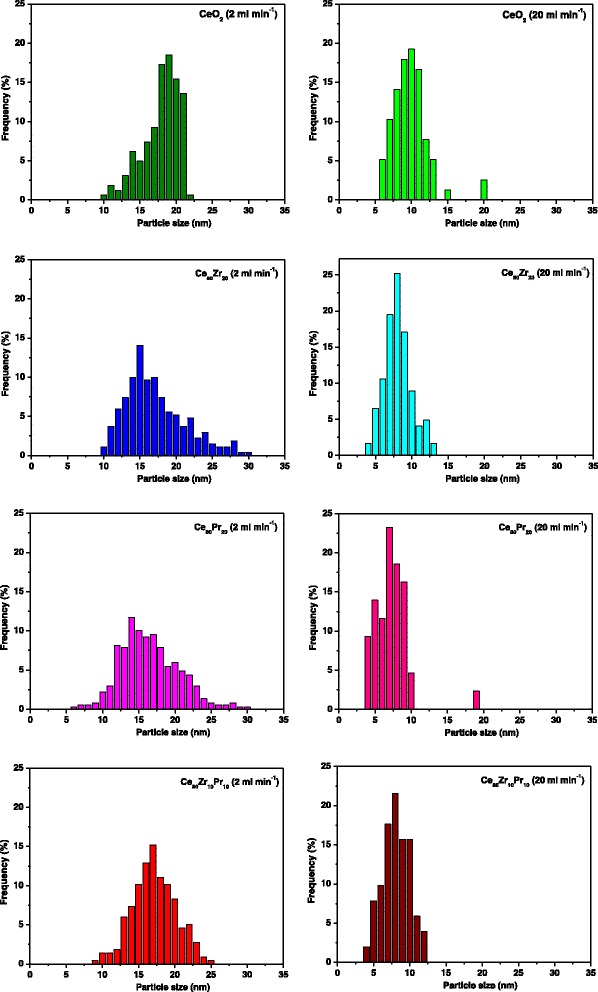
Size distributions of the primary particles derived by FESEM analysis

Electron microscopy analysis was used to find out the morphological properties of the catalysts. As shown in Fig. 4, the samples exhibit agglomerates of primary particles with dimensions of ca. 10–20 nm. In order to compare the particle size distributions of the catalysts obtained with the MVIR using two inlet flow rates, a detailed analysis of the micrographies has been performed. Figure 5 shows the results of the granulometric analysis carried out by FESEM analysis: it was found that the dimension of the primary particles was considerably reduced by increasing the inlet flow rate. In fact, faster mixing induces higher super-saturation locally in the region where the inlets collide, which in turn increases the nucleation rate of the primary particles. High nucleation rates increase the number of primary particles being formed, which are the nuclei over which the metal precursors diffuse and concur to particle growth. This leads to comparatively smaller primary particles than in conventional precipitation processes, because of the high number of involved nuclei [20, 25]. Hence, small primary particles indicate a higher degree of mixing at all scales (including the molecular level which is the most difficult to achieve especially in liquid systems), as verified in [28] with equivalent geometry.

The simulation of the fluid-dynamic regime computed in terms of mixture fraction of the reactants at the macro-scale, revealed that 2 ml min^−1^ entails a heterogeneous concentration distribution in the volume of the mixing chamber [28]. On the other hand, the macro-mixing is very effective at 20 ml min^−1^, thus reaching a flat profile of mean mixture fraction close to the mixing chamber exit in both conditions. At the micro-scale, i.e., the molecular level mixing, the turbulence intensity at 20 ml min^−1^ provides extremely low segregation [28], which fosters super-saturation and thus nucleation phenomena.

**Fig. 6:**
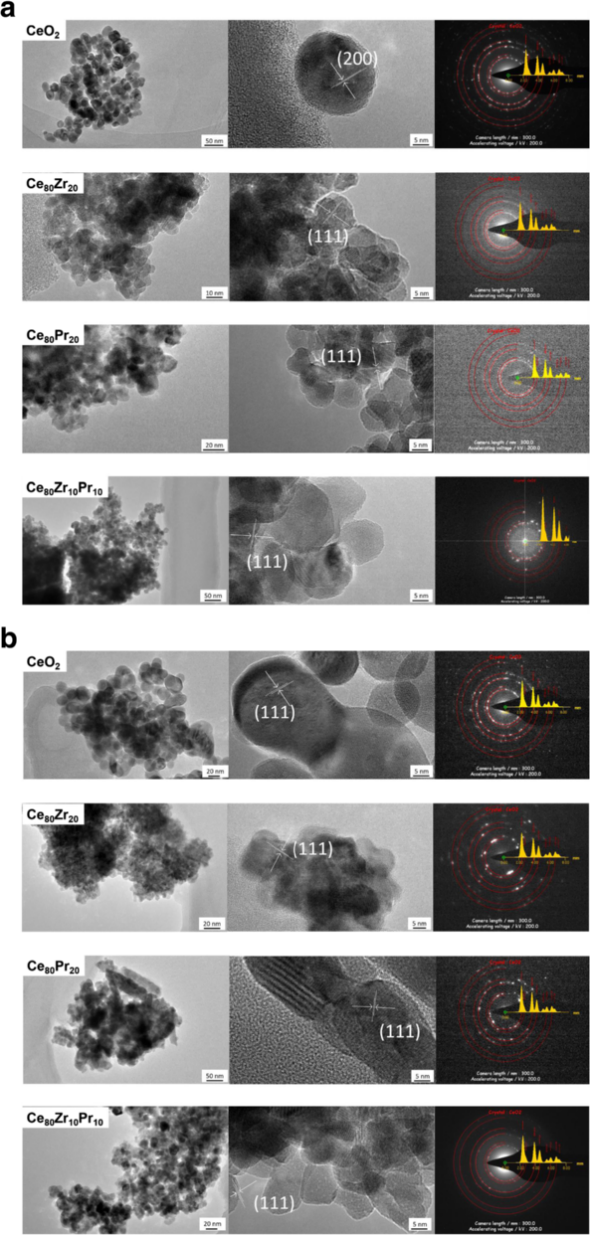
HR-TEM images of the prepared catalysts (**a** 2 ml min^−1^ and **b** 20 ml min^−1^): low and high magnification images (*right* and *center*) and electron diffraction (*right*)

It is worth mentioning that, contrary to primary particle size (strictly depending on the syntheses conditions in the MIVR), there is no direct correlation between the final aggregate size and the flow conditions in the reactor was attempted, since the aggregation phenomena occur prevalently in the beaker receiving the solution and in the subsequent centrifuging and filtering steps. In this regard, HR-TEM measurements were performed in order to better observe the primary particles nature as well as to clarify the way the single primary particles aggregate. TEM was also used to estimate the crystal size of the particles in the different systems. The results are reported in Fig. 6. From TEM images, it is put in evidence for the CeO_2_ samples containing Zr and/or Pr ion metals, the extreme tendency of the single crystals to aggregate in big clusters (around hundreds of nanometers—see left images of Fig. 6). HRTEM evidences the good crystallinity of the different catalysts; in particular, for each sample, a single crystal has been indexed (Fig. 6, right). Electron diffraction confirms what found with XRD, showing fully crystalline and randomly oriented samples.

Finally, energy dispersive spectroscopy (EDS) have been performed to estimate the stoichiometry of the different samples. EDS microanalysis from FESEM was performed in different regions of each sample (five spots), resulting on an average atomic composition of the catalysts. Although EDS is a semi-quantitative analysis, it provides a bulk information, which is useful in this case. It emerges that the change in the inlet flow rate, and consequently the different mixing process of the reagents, has an important role in determining the final composition of the particles. The results, referred to spot regions 20 μm wide, are gathered in Table 2 and confirm that the real amounts of dopants are close the ones expected nominally, especially for the ceria-zirconia samples. Also the ternary oxides report a satisfactory Zr and Pr incorporation, although at 2 ml min^−1^ the former is below the nominal value of 10 %, and the latter is above it. The only exception is the sample Ce_80_Pr_20_ at 20 ml min^−1^, which is much richer in Pr than expected, suggesting that probably Pr oxide precipitation occurs faster cerias, and so higher supersaturation occurring at 20 ml min^−1^ hinders Pr incorporation in CeO_2_ lattice, promoting instead the pure oxide formation.

**Table 2:** Ce, Zr, and Pr atomic percentages estimated by EDS

Sample	MIVR single channel inlet flow rate = 2 ml min^−1^			MIVR single channel inlet flow rate = 20 ml min^−1^		
	Ce (%)	Zr (%)	Pr (%)	Ce (%)	Zr (%)	Pr (%)
CeO_2_	100	–	–	100	–	–
C_80_Zr_20_	81	19	-	82	18	–
Ce_80_Pr_20_	79	–	21	63	–	37
Ce_80_Zr_10_Pr_10_	81	5	14	83	7	10

Micro-analyses from HR-TEM images were also performed, and reported in Fig. 6 (Cu and C refer to the lacey carbon coated copper grid; Co and Fe to the magnets). The results essentially confirm the ones of the EDS reported in Table 2, but are referred to a much smaller spot (around 50 × 50 nm), thus confirming a rather satisfactory composition homogeneity.

**Fig. 7:**
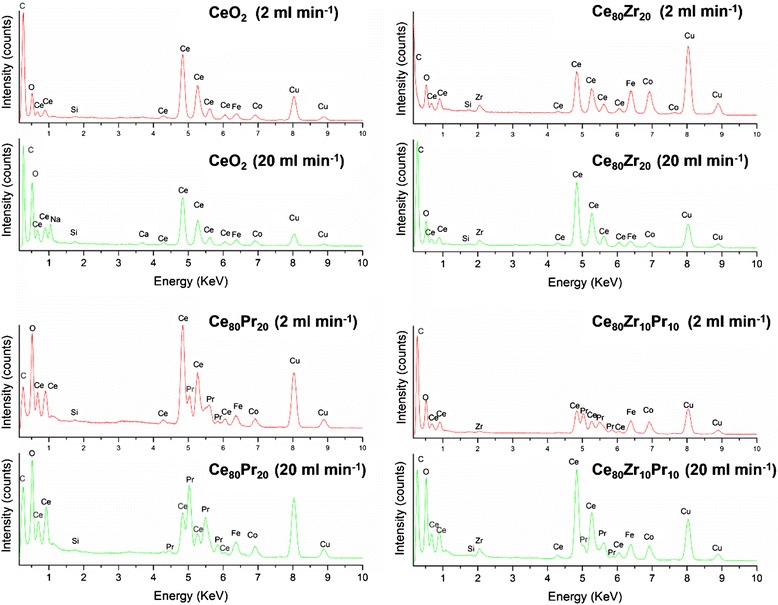
EDS micro-analysis of the prepared catalysts with HR-TEM (*Cu* and *C* refer to the lacey carbon coated copper grid; *Co* and *Fe* to the magnets)

Overall, these results prove that the fast precipitation occurring in MIVR is able to incorporate dopants such as Zr and Pr, up to the amounts investigated in this paper, and that micro-scale turbulence intensity has to be tuned to attain the proper doping amounts (Fig. 7).

### Structural Defects of the Catalysts

**Fig. 8:**
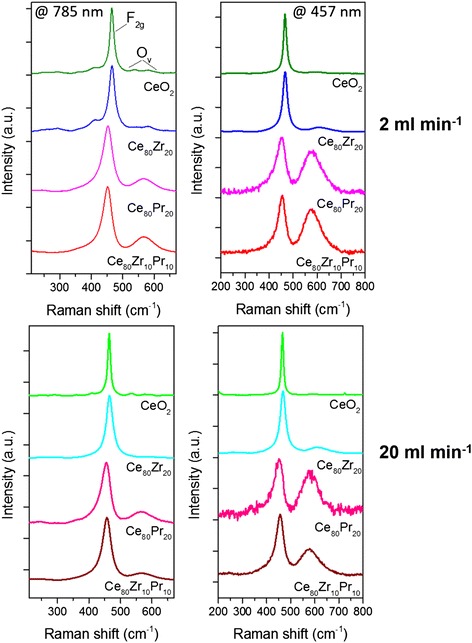
Raman spectra of the catalysts acquired at 785 nm (*left*) and 457.9 nm (*right*) excitations for samples prepared at 2 ml min^−1^ (*top*) and 20 ml min^−1^ (*bottom*) inlet flow rates

Raman spectra of the samples were collected in order to check the presence of oxygen vacancies and the relative concentration, which was shown to be correlated to the catalytic activity [29]. The measurements performed with a 785- and 457.9-nm laser excitation are reported in Fig. 8 for the different materials synthesized at 2 and 20 ml min^−1^ inlet flow rates. The higher energy laser is expected to provide a higher surface sensitivity, due to the lower penetration depth into the sample, while the lower energy excitation line carries overall information concerning also the inner volume of the particles. The spectral region between 200 and 800 cm^−1^ is dominated by an intense band, assigned to the Ce-O_8_ crystal unit vibration (F_2g_ mode of the fluorite lattice) [30]. For pure CeO_2_ and Ce_80_Zr_20_, such band is centered around 465 cm^−1^, while a red-shift towards 453 cm^−1^ is observed for Pr-containing compounds. Several lower intensity bands are detected on both sides of the main peak. In particular, two peaks can be resolved at higher Raman shifts, around 580 and 601 cm^−1^ for CeO_2_ and Ce_80_Zr_20_, at 785 nm, which are usually observed as a single convoluted band for more energetic excitation wavelengths [27, 29]. These peaks arise from Frenkel type anion defects, in which an oxygen ion occupies an interstitial position, leaving a vacancy at its original lattice site [31]. A single broad band centered around 567 cm^−1^ is observed for Pr-doped ceria, which was previously suggested to be the convolution of three contributions due to Pr-induced (~545 and ~570 cm^−1^) and intrinsic oxygen vacancies (~595 cm^−1^) [32].

The concentration of vacancies can be evaluated through the ratio between the area of the defect-induced and the F_2g_ bands, O_v_/F_2g_ [29]. The calculated values are reported in Table 3 for the two excitation wavelengths. The trends are similar and the defect density increases in the following order:

**Table 3:** O_v_/F_2g_ vibrational bands ratios calculated from the Raman spectra

Sample	MIVR single channel inlet flow rate = 2 ml min^−1^		MIVR single channel inlet flow rate = 20 ml min^−1^	
	457.9 nm	785 nm	457.9 nm	785 nm
CeO_2_	0.25	0.29	0.11	0.16
C_80_Zr_20_	0.31	0.29	0.37	0.14
Ce_80_Pr_20_	1.09	0.33	1.05	0.83
Ce_80_Zr_10_Pr_10_	1.23	0.40	0.79	0.22

$$ \mathrm{C}\mathrm{e}{\mathrm{O}}_2 < \mathrm{C}{\mathrm{e}}_{80}\mathrm{Z}{\mathrm{r}}_{20} < \mathrm{C}{\mathrm{e}}_{80}\mathrm{Z}{\mathrm{r}}_{10}\mathrm{P}{\mathrm{r}}_{10} < \mathrm{C}{\mathrm{e}}_{80}\mathrm{P}{\mathrm{r}}_{20} $$

A higher oxygen vacancies concentration is therefore attained by the addition of dopant elements. Interestingly, because of the small size of the nanoparticles, it is difficult to observe differences in the Raman spectra at the two excitation wavelengths in case of CeO_2_ and Ce_80_-Zr_20,_ while for the Pr containing catalyst a boost of the defect band, and thus of the O_v_/F_2g_ value, occurs upon excitation at 457.9 nm. The effect is due to the relevant absorption of Pr-doped ceria in the visible range, which limits both the penetration depth of the incident radiation and as a consequence the collected scattering signal to the most superficial layer of the sample, suggesting that the oxygen vacancies and therefore the dopant concentration are higher at the surface [33].

### Catalytic Activity Tests

**Fig. 9:**
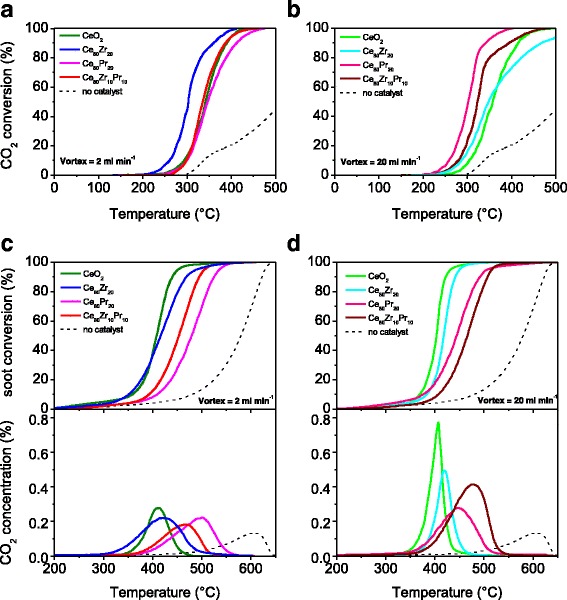
CO to CO_2_ conversion versus temperature over the fresh catalysts (sections a and b). Soot conversion and CO_2_ concentration profiles (sections c and d) over the same catalysts, under “tight” contact

The catalytic behavior towards CO oxidation was studied in the 100–500 °C temperature range. Figure 9 summarizes the CO conversion values (%) as a function of the temperature for the catalysts synthesized with flow rates of 2 ml min^−1^ (section a) and 20 ml min^−1^ (section b), respectively. As a whole, all the catalysts improve greatly the CO conversion, whereas only ca. 40 % of CO converts naturally to CO_2_ at 500 °C (uncatalized reaction). Among the set of catalysts synthesized with inlet flow rates of 2 ml min^−1^, the Ce_80_Zr_20_ sample outperforms the other oxides, exhibiting comparable performances (Table 4). The high activity for this catalyst is the result of the best compromise among the structural defects, redox-active centres (Ce^3+^/Ce^4+^ pairs) and small particle size [6, 34]. On the other hand, for the catalysts obtained at higher inlet flow rates, the following increasing order of activity (in terms of T_10%_ and T_50%_) can be drawn:

**Table 4:** CO oxidation activity results over the fresh catalysts

Sample	MIVR single channel inlet flow rate = 2 ml min^−1^			MIVR single channel inlet flow rate = 20 ml min^−1^		
	T_10%_	T_50%_	T_90%_	T_10%_	T_50%_	T_90%_
CeO_2_	288	342	391	305	359	417
Ce_80_Zr_20_	253	303	360	286	347	467
Ce_80_Pr_20_	295	346	410	258	301	344
Ce_80_Zr_10_Pr_10_	295	334	387	278	324	397

$$ \mathrm{C}\mathrm{e}{\mathrm{O}}_2 < \mathrm{C}{\mathrm{e}}_{80}\mathrm{Z}{\mathrm{r}}_{20} < \mathrm{C}{\mathrm{e}}_{80}\mathrm{Z}{\mathrm{r}}_{10}\mathrm{P}{\mathrm{r}}_{10} < \mathrm{C}{\mathrm{e}}_{80}\mathrm{P}{\mathrm{r}}_{20} $$

(Table 4 reports the T_10%_, T_50%_, and T_90%_ values). In the present case, the catalysts exhibit similar crystal sizes (in the 7.6–10.4 nm range), except for the CeO_2_ (=14.5 nm), and then it is possible to observe a direct correlation between the O_v_/F_2g_ intensity ratios (Raman spectra recorded by exciting at 457.9 nm) and the catalytic performance for the CO oxidation (Fig. 10). This means that structural (superficial) defects play a key role for the CO oxidation reaction, despite the small crystal size may have a beneficial effect on the oxidation activity. At higher conversions (T_90%_), indeed, mass-transport phenomena play a major role, thus masking the effective kinetic effects.

**Fig. 10:**
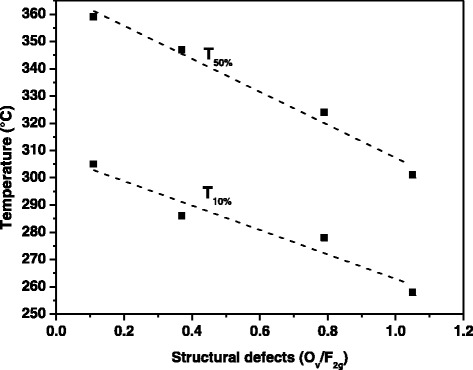
Correlation between the O_v_/F_2g_ intensity ratios of the catalysts (Vortex = 20 ml min^−1^) and their catalytic performance for the CO oxidation (in terms of T_10%_ and T_50%_ values). The O_v_/F_2g_ intensity ratios have been obtained using the Raman spectra recorded by exciting at 457.9 nm

The soot oxidation activity has been examined in the 200–650 °C temperature range. Figure 9 (sections c and d) shows the soot conversion values (%) as a function of the temperature achieved for the catalysts (in “tight” soot-catalyst contact) along with Printex-U (no catalyst). For the set of catalysts obtained at low inlet flow rate, the increasing order of soot oxidation activity is:

$$ \mathrm{C}{\mathrm{e}}_{80}\mathrm{P}{\mathrm{r}}_{20} < \mathrm{C}{\mathrm{e}}_{80}\mathrm{Z}{\mathrm{r}}_{10}\mathrm{P}{\mathrm{r}}_{10} < \mathrm{C}{\mathrm{e}}_{80}\mathrm{Z}{\mathrm{r}}_{20} \approx \mathrm{C}\mathrm{e}{\mathrm{O}}_2 $$

whereas for the counterpart (MIVR inlet flow rates = 20 ml min^−1^), the increasing activity order is:

$$ \mathrm{C}{\mathrm{e}}_{80}\mathrm{Z}{\mathrm{r}}_{10}\mathrm{P}{\mathrm{r}}_{10} < \mathrm{C}{\mathrm{e}}_{80}\mathrm{P}{\mathrm{r}}_{20} < \mathrm{C}{\mathrm{e}}_{80}\mathrm{Z}{\mathrm{r}}_{20} < \mathrm{C}\mathrm{e}{\mathrm{O}}_2 $$

Table 5 summarizes the T_10%_, T_50%_ and T_90%_ values.

**Table 5:** Soot oxidation activity results over the fresh catalysts

Sample	MIVR single channel inlet flow rate = 2 ml min^−1^			MIVR single channel inlet flow rate = 20 ml min^−1^		
	T_10%_	T_50%_	T_90%_	T_10%_	T_50%_	T_90%_
CeO_2_	360	409	440	367	405	422
Ce_80_Zr_20_	350	433	561	381	416	443
Ce_80_Pr_20_	412	478	527	374	443	492
Ce_80_Zr_10_Pr_10_	395	454	495	405	465	506

As expected, the CO_2_ concentration (%) profiles reflect the same activity orders for the prepared catalysts. These results confirm that dopants like Zr and Pr species do not promote the soot oxidation reaction over these “unstructured” ceria-based catalysts, at variance of the nanostructured catalysts (e.g., Ce–Zr–O nanopolyhedra, Ce–Pr–O nanocubes) described in our previous studies [12, 16, 35, 36]. The incorporation of Zr and Pr species, indeed, increases the presence of structural defects (see Table 3) but decreases the surface redox centers, which are directly dependent on the Ce^3+^ - Ce^4+^ surface density [16]. Indeed, both the CeO_2_ catalysts resulted the most active soot oxidation catalysts among those synthesized with the MVIR technique.

As a whole, defect sites and oxygen vacancies are among the most desirable active sites for oxidation catalysts, since they modify the Fermi Energy level thus favoring oxidation reactions [16]. However, in the present case, the structural defects do not have a beneficial role on the soot oxidation reaction; it should be pointed out that the feasibility of the soot combustion mechanism depends to a great extent on the interaction between the soot particles and the catalyst surface [35, 37].

## Conclusions

In the present work, we investigated a set of ceria-based catalysts (namely, Ce–Zr–Pr oxides, Ce–Zr oxides, Ce–Pr oxides and pure CeO_2_) prepared by the multi-inlet vortex reactor (MIVR).

As a whole, when the catalysts exhibit comparable crystallite sizes, a direct correlation between the O_v_/F_2g_ vibrational band intensity ratios (Raman spectra recorded by exciting at 457.9 nm, more sensitive to the nanoparticles surface) and the catalytic performance for the CO oxidation (in terms of T_10%_ and T_50%_) occurs. This confirms that the structural (superficial) defects on solid surfaces, as measured by the Raman spectroscopy, play a key role for the CO oxidation in the 150–500 °C temperature range. Specifically, the higher the concentration of oxygen vacancies the higher the surface reactivity. Moreover, it has been observed that smaller particles promote the CO oxidation. The incorporation of Zr and Pr dopants in the ceria lattice increases the amount of surface defects and then the reactivity for the CO oxidation, in the order CeO_2_ < Ce_80_Zr_20_ < Ce_80_Zr_10_Pr_10_ < Ce_80_Pr_20_. On the other hand, the presence of zirconium and praseodymium into the ceria does not have a direct beneficial role on the soot oxidation for these “unstructured” materials, at variance of the nanostructured ones (e.g., Ce–Zr–O nanopolyhedra, Ce-Pr-O nanocubes), previously investigated by our research group. In the present case, the most active catalysts resulted to be pure ceria synthesized at either 2 ml min^−1^ or 20 ml min^−1^. Thus, the oxygen vacancies do not exhibit a direct beneficial effect on the soot oxidation reaction for these ceria-based materials, thus showing a different catalytic behavior for the two oxidation reactions.

## References

[1] Terribile D, Llorca J, Boaro M, de Leitenburg C, Dolcetti G, Trovarelli A. Fast oxygen uptake/release over a new CeO_x_ phase. Chem Commun. 1998;1897–1898

[2] Aneggi E, Boaro M, de Leitenburg C, Dolcetti G, Trovarelli A (2006). Insight into the redox properties of ceria-based oxides and their implications in catalysis. J Alloys Compd.

[3] Trovarelli A, Fornasiero P (2013). Catalysis by ceria and related materials.

[4] Aneggi E, Llorca J, Boaro M, Trovarelli A (2005). Surface-structure sensitivity of CO oxidation over polycrystalline ceria powders. J Catal.

[5] Wu Z, Li M, Overbury SH (2012). On the structure dependence of CO oxidation over CeO_2_ nanocrystals with well-defined surface planes. J Catal.

[6] Piumetti M, Bensaid S, Fino D, Russo N (2016). Ceria-zirconia nanocatalysts for CO oxidation: study on surface properties and reactivity. Appl Catal B.

[7] Piumetti M, Bensaid S, Russo N, Fino D (2015). Nanostructured ceria-based catalysts for soot combustion: investigations on the surface sensitivity. Appl Catal B.

[8] Aneggi E, Wiater D, de Leitenburg C, Llorca J, Trovarelli A (2014). Shape-dependent activity of ceria in soot combustion. ACS Catal.

[9] Miceli P, Bensaid S, Russo N, Fino D (2014). CeO_2_-based catalysts with engineered morphologies for soot oxidation to enhance soot-catalyst contact. Nanoscale Res Lett.

[10] Bensaid S, Russo N, Fino D (2013). CeO_2_ catalysts with fibrous morphology for soot oxidation: the importance of the soot-catalyst contact conditions. Catal Today.

[11] Bensaid S, Caroca CJ, Russo N, Fino D (2011). Detailed investigation of non catalytic DPF regeneration. Can J Chem Eng.

[12] Andana T, Piumetti M, Bensaid S, Russo N, Fino D, Pirone R (2016) Nanostructured ceria-praseodymia catalysts for diesel soot combustion. Appl Catal B. Environ 197:125–137

[13] Yang Z, Zhou K, Liu X, Tian Q, Lu D, Yang S (2007). Single-crystalline ceria nanocubes: size-controlled synthesis, characterization and redox property. Nanotechnology.

[14] Mai H-X, Sun L-D, Zhang Y-W, Si R, Feng W, Zhang H-P, Liu H-C, Yan C-H (2005). Shape-selective synthesis and oxygen storage behavior of ceria nanopolyhedra, nanorods, and nanocubes. J Phys Chem B.

[15] Royer S, Duprez D (2011). Catalytic oxidation of carbon monoxide over transition metal oxides. ChemCatChem.

[16] Piumetti M, Bensaid S, Russo N, Fino D (2016). Investigations into nanostructured ceria–zirconia catalysts for soot combustion. Appl Catal B.

[17] Bueno-López A (2014). Diesel soot combustion ceria catalysts. Appl Catal B.

[18] Atribak I, López-Suárez FE, Bueno-López A, García-García A (2011). New insights into the performance of ceria-zirconia mixed oxides as soot combustion catalysts. Identification of the role of “active oxygen” production. Catal Today.

[19] Andana T, Piumetti M, Bensaid S, Russo N, Fino D, Pirone R (2016). CO and soot oxidation over Ce-Zr-Pr oxide catalysts. Nanoscale Res Lett.

[20] Palanisamy B, Paul B (2012). Continuous flow synthesis of ceria nanoparticles using static T mixers. Chem Eng Sci.

[21] Thakur RK, Vial C, Nigam KDP, Nauman EB, Djelveh G (2003). Static mixers in the process industries—a review. Chem Eng Res Dev.

[22] Lince L, Marchisio DL, Barresi AA (2011). A comparative study for nanoparticle production with passive mixers via solvent-displacement: use of CFD models for optimization and design. Chem Eng Proc.

[23] Gradl J, Schwarzer HC, Schwertfirm F, Manhart M, Peukert W (2006). Precipitation of nanoparticles in a T-mixer: coupling the particle population dynamics with hydrodynamics through direct numerical simulation. Chem Eng Process.

[24] Santillo G, Deorsola FA, Bensaid S, Russo N, Fino D (2012). MoS_2_ nanoparticle precipitation in turbulent micromixers. Chem Eng J.

[25] Liu Y, Cheng JC, Prud’homme RK, Fox RO (2008). Mixing in a multi-inlet vortex mixer (MIVM) for flash nano-precipitation. Chem Eng Sci.

[26] Laha SC, Ryoo R. Synthesis of thermally stable mesoporous cerium oxide with nanocrystalline frameworks using mesoporous silica templates. Chem Commun. 2003;2138–2139. 10.1039/b305524h 13678169

[27] Filtschew A, Hofmann K, Hess K. Ceria and its defect structure: new insights from a combined spectroscopic approach. J. Phys. Chem. C. 2016; doi: 10.1021/acs.jpcc.6b00959

[28] Bensaid S, Deorsola FA, Marchisio DL, Russo N, Fino D (2014). Flow field simulation and mixing efficiency assessment of the multi-inlet vortex mixer for molybdenum sulfide nanoparticle precipitation. Chem Eng J.

[29] Li Y, Wei Z, Gao F, Kovarik L, Baylon AL, Peden CHF, Wang Y (2015). Effect of oxygen defects on the catalytic performance of VOx/CeO2 catalysts for oxidative dehydrogenation of methanol. ACS Catal.

[30] McBride JR, Hass KC, Poindexter BD, Weber WH (1994). Raman and X-ray studies of Ce1 − xRexO2 − y, where Re = La, Pr, Nd, Eu, Gd, and Tb. J Appl Phys.

[31] Agarwal S, Zhu X, Hensen EJM, Lefferts L, Mojet BL (2014). Defect chemistry of ceria nanorods. J Phys Chem C.

[32] Westermann A, Geantet C, Vernoux P, Loridant S. Defects band enhanced by resonance Raman effect in praseodymium doped CeO2. J Raman Spectrosc. 2016, doi: 10.1002/jrs.4943.

[33] Gao M, Lu J, Wu Y, Wang Y, Luo M (2011). UV and visible Raman studies of oxygen vacancies in rare-earth-doped ceria. Langmuir.

[34] Piumetti M, Andana T, Bensaid S, Russo N, Fino D, Pirone R (2016). Study on the CO oxidation over ceria-based nanocatalysts. Nanoscale Res Lett.

[35] Fino D, Bensaid S, Piumetti M, Russo N (2016). A review on the catalytic combustion of soot in diesel particulate filters for automotive applications: from powder catalysts to structured reactors. Appl Catal A.

[36] Piumetti M, Andana T, Bensaid S, Fino D, Russo N, Pirone R. Ceria-based nanomaterials as catalysts for CO oxidation and soot combustion: effect of Zr-Pr doping and structural properties on the catalytic activity. AICHe J. (submitted).

[37] Bensaid S, Russo N (2011). Low temperature DPF regeneration by delafossite catalysts, Catal. Today.

